# Peptides From Animal and Fishery Byproducts: Uplifting the Functionality of Fifth Quarter

**DOI:** 10.1002/fsn3.70140

**Published:** 2025-04-18

**Authors:** Akhilesh Kumar Verma, Pramila Umaraw, Pavan Kumar, Nitin Mehta, Neelesh Sharma, Devendra Kumar, Awis Qurni Sazili, Sung‐Jin Lee

**Affiliations:** ^1^ Department of Livestock Products Technology, College of Veterinary and Animal Sciences Sardar Vallabhbhai Patel University of Agriculture and Technology Meerut Uttar Pradesh India; ^2^ Department of Livestock Products Technology, College of Veterinary Science Guru Angad Dev Veterinary and Animal Sciences University Ludhiana Punjab India; ^3^ Division of Veterinary Medicine, Faculty of Veterinary Sciences and Animal Husbandry Sher‐e‐Kashmir University of Agricultural Sciences and Technology of Jammu Ranbir Singh Pura Union Territory of Jammu and Kashmir India; ^4^ Division of Livestock Products Technology Indian Veterinary Research Institute Bareilly Uttar Pradesh India; ^5^ Department of Animal Science, Faculty of Agriculture Universiti Putra Malaysia Serdang Malaysia; ^6^ Halal Product Research Institute Universiti Putra Malaysia Serdang Malaysia; ^7^ Department of Applied Animal Science, College of Animal Life Sciences Kangwon National University Chuncheon Republic of Korea

**Keywords:** antimicrobial, antioxidant, functional activity, packaging, peptides, sensory attributes

## Abstract

The meat and fish sectors are primary sources of animal protein for human consumption. However, they also generate large volumes of byproducts and organic waste annually, posing major challenges in terms of sustainable disposal. These byproducts have the potential to be repurposed into high‐value, low‐volume products, such as bioactive peptides or hydrolysates. Various methods used for the recovery of bioactive peptides from meat byproducts are enzymatic hydrolysis, microbial fermentation, ultrasonic‐assisted, pulsed electric field, high hydrostatic pressure, microwave‐assisted, and subcritical water processing. These bioactive peptides possess various functional properties, including antioxidant, antimicrobial, and antihypertensive effects. Incorporating them into food products could enhance both the functionality and quality of these products. In light of growing consumer demand for natural, eco‐friendly ingredients, as well as sustainable practices in food production and packaging, the generation and use of bioactive peptides and hydrolysates offer a promising strategy. This approach not only mitigates environmental challenges but also fosters the sustainable growth of the meat and fishery industries, ensuring long‐term ecological and economic viability. This review explores new opportunities and avenues for utilizing animal and marine byproducts in producing bioactive peptides and their potential applications in meat products and processing techniques, thereby supporting the sustainable growth of the meat and fishery industries.

## Graphical abstract

Recovery of peptide reduces pollution, increases return, and functionality of products. Meat byproducts and processing techniques raise the sustainability of the meat industry. These peptides can also be used as active components of the packaging system. Umami peptides are used as taste enhancers for the development of new products.

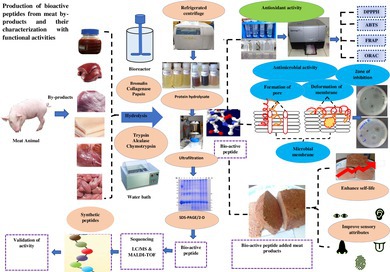

## 1 Introduction

Animal byproducts are all parts of an animal that are not included in a dressed carcass such as blood, offals (edible and inedible), fats, hides, and skin. These non‐meat parts are often called as “fifth quarter” as they comprise about 55% of the live weight of cattle and about 48% of the live weight of pigs (Drummond et al. 2019). According to Jayathilakan et al. (2012), byproducts may contribute up to 44% of the live weight of cattle and if utilized efficiently, it might enhance the gross income by 11.4% (Jayathilakan et al. 2012). The byproducts or the coproducts of animal slaughtering can be either edible or inedible. The edible byproducts are known as offal meat, variety meat or fancy meat and are used for human consumption in various parts of the world, but have a niche market for consumption. The inedible byproducts comprise hides and skins, horns, hoofs, trimmings, viscera, and gastrointestinal tract (GIT) content.

These byproducts have long been used in the production of animal/poultry/fish/aquaculture feed. But with the emergence of zoonotic diseases like bovine spongiform encephalitis (BSE), foot and mouth disease (FMD), avian influenza, swine fever, and others, these biological materials from animals have been classified into three categories according to their hazard potential by European Legislation (EC) 1069/2009. Classes 1 and 2 are high‐specified risk materials (SRM) while class 3 materials are obtained from healthy animals but not intended for consumption (Drummond et al. 2019). The European Fat Processors and Renderers Association (EFPRA) reported the generation of 5 million tons of classes 1 and 2 byproducts and 12 million tons of class 3 byproducts from meat processing plants (Bechaux et al. 2019). A huge amount of class 3 byproducts leaves the slaughterhouse as class 1, degrading its potential and increasing the disposal cost (Henchion and McCarthy 2019). These are directed toward the non‐food category even though they contain high‐quality protein with high nutraceutical and food potential for human use. Utilization of even half of it would generate 200 million kg of more protein per year with an approximate value of $200 million annually in the United States alone (Henchion and McCarthy 2019). Similarly, the marine byproducts, which consist of fillet, round, eviscerated, or beheaded fish from fresh catch and skin, fins, head, trimmings, viscera, frames, substandard muscles, trimmings, shellfish and crustacean shell waste, and roe from the processing industry, are also underutilized. According to FAO estimates, approximately 25% of the catch and nearly 50% of fish are discarded during processing, resulting in the generation of around 106 million tons of marine waste each year (Santos et al. 2020). Most of these protein‐rich class 3 byproducts and marine processing byproducts are converted into animal feed and/or fertilizers (high volume, low value) which can be directed toward more profitable sources of functional components (low volume, high value) such as bioactive hydrolysates and/or peptides.

Bioactive peptides (BP) are small chain amino acids produced by the degradation of larger protein molecules under external or internal action. These are defined as peptide sequences within a protein that exert a positive effect on human health or body functions in addition to their nutritional value (Przybylski et al. 2016). These are small chains that range from 2 to 20 amino acid residues per molecule. These bioactive peptides regulate the functionality of systems by targeting the biomarkers. Their activity is largely governed by their amino acid composition, length, sequence, spatial arrangement, and charge. BP has wide applications in various industries such as pharmacology, cosmetology, therapeutics, nutraceuticals, and food.

The demand for bioactive peptides (BPs) and related products is experiencing rapid growth, with a projected compound annual growth rate (CAGR) of 8.2% from 2020 to 2027, reaching an estimated market value of $88.3 billion. This surge is primarily driven by increasing consumer awareness of health and nutrition, rising fitness activity, and the growing demand for dietary supplements, functional foods, therapeutics, and nutraceuticals (Santos et al. 2020). In 2019, the functional food sector led the bioactive peptide market, accounting for 35.6% of the total revenue generated (Santos et al. 2020).

Animal byproducts or coproducts are important sources for the generation of bioactive peptides, as the raw material is abundantly generated during the processing of meat and seafood, which are widely wasted, causing an environmental burden. Generating BP from coproducts would augment sustainable growth and development, which is the need of the hour. Thus, the present review critically summarizes the various technological aspects of the efficient utilization of byproducts and their use in meat processing as natural bioactive compounds by reducing the disposal burden of the industry and expanding the scope of natural bioactive peptides.

## 2 Generation of Bioactive Peptides

**FIGURE 1:**
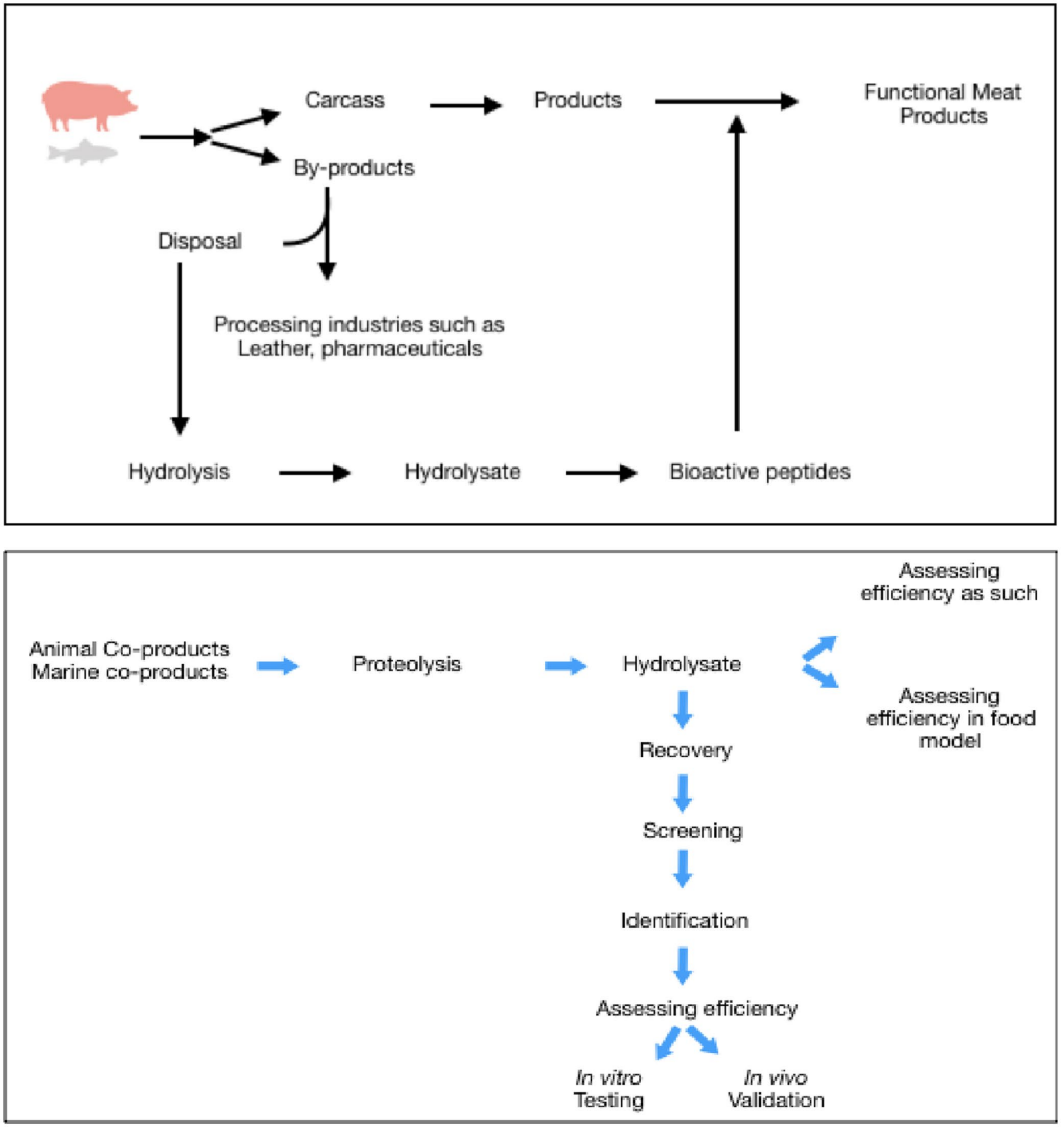
(a, b) Generation of bioactive peptides.

Bioactive peptides can only be generated from proteins by the application of external factors such that they bring about structural change and/or reduce their size or fractioning by hydrolysis (Figure 1). Enzyme hydrolysis is the most popular method to generate peptides from protein sources. With increasing awareness about the beneficial effects of BP, there has been more focus on the identification of novel BP. Although most of the available bioactive peptides have been identified and maintained in silico, such as BIOPEP, PepBank, EROP‐Moscow, and APD, the search for novel BP with newer functionality from different sources is still continuing. Animal or marine coproducts that are produced in large quantities during processing are a promising source for the generation of bioactive peptides, as they are rich in proteins. Bioactive peptides from animal or marine coproducts can be generated by protein hydrolysis using enzymes, microbial fermentation, and/or acid–alkali treatment.

### 2.1 Enzymes

Standard pH and temperature are the primary requirements of commonly used enzymes for the hydrolysis of proteins. Animal‐origin proteases such as pepsin, trypsin, and chymotrypsin are commonly used in producing hydrolysates. Jurado et al. (2012) assayed three methods: dissolution of pepsinogen, dissolution followed by coagulation, and dissolution, coagulation, and lyophilization for extraction of pepsin from pig stomach. Consequent concentration by lyophilization increased the purity and decreased the storage space, which is now a popularly used method of pepsin extraction. Pepsin with an activity of 526.5 U/mL was extracted from catfish gastric using tris‐buffer, and that from milkfish gastric reported 397 U/mL (milkfish) activity (Nurhayati et al. 2020). Similarly, trypsin from albacore tuna liver was prepared by Sripokar et al. (2019) and used for the preparation of protein hydrolysate with functional properties.

Fungal enzyme (protease P “Amano”) was used to generate protein hydrolysate from sheep viscera (stomach and intestines), a coproduct of mutton production. A 34% degree of hydrolysis was obtained from 1% w/w enzyme produced with more than 64% protein recovery at optimum conditions such as temperature 43°C + 1°C at pH 7.0 for 45 min. Similarly, protein hydrolysate from chicken liver with good antioxidant (1.13 μg ascorbic acid equivalent (AAE) mg^−1^ proteins) and antimicrobial activity against *Micrococcus luteus* (12 mm diameter zone of inhibition) was obtained by commercial protease, Alcalase 2.5 (Chakka et al. 2015). Alcalase produced hydrolysate with better iron‐binding capacity than trypsin, flavourzyme, and neutrase from byproducts of mackerel and scad processing (Chew et al. 2019; Wang et al. 2013). Alcalase hydrolysate has shown better calcium‐binding capacity than those produced by papain or flavourzyme.

The degree of hydrolysis is a time and enzyme‐dependent phenomenon that is positively correlated. In a study on porcine blood hydrolysates obtained with enzymes alcalase, trypsin, and papain, Verma et al. (2019a) reported the highest degree of hydrolysis after 6 h of activity for alcalase and papain, but for trypsin, the highest degree of hydrolysis was reported after 4 h, which later increased insignificantly. The study also revealed that the functional activity of hydrolysates is positively correlated to the degree of hydrolysis, as the hydrolysates achieved at 6 h intervals showed significantly better 2,2′‐azino‐bis(3‐ethylbenzothiazoline‐6‐sulfonic acid (ABTS) inhibition, 2,2‐diphenyl‐1‐picrylhydrazyl (DPPH) inhibition, and ferric reducing antioxidant power (FRAP) values.

Trypsin hydrolysate produced from shrimp processing coproducts also evinced superior calcium‐binding capacity over alcalase, pepsin, flavourzyme, or protamex hydrolysates. Commercial peptides alcalase, flavourzyme, neutrase, pepsin, protamex, and trypsin produced similar degrees of hydrolysis but peptic hydrolysate evinced better DPPH radical scavenging activity (1.63 mg/mL) than others (Ahn et al. 2014). Similarly, bioactive peptides with antibacterial activity against *Bacillus megaterium* , *Yersinia rockery*, and *Edwardsiella tarda* were obtained from protamex hydrolysis of white shrimp coproducts (Robert et al. 2014). Trypsin, alcalase, and papain produced 26.82%, 23.56%, and 19.12% degrees of hydrolysis from the porcine liver. Trypsin evinced the highest degree of hydrolysis as well as superior anti‐oxidative ABTS radical scavenging activity (86.79%; FRAP 14.92%) and antimicrobial potential (zone of inhibition against *L. monocytogenes* (23.39 ± 0.31 mm) and moderate inhibition against *S. aureus* (18.35 ± 0.65 mm) and *E. coli* (18.17 ± 0.36 mm)) than others (Verma, Chatli, Kumar, et al. 2017).

### 2.2 Microbial Fermentation

Fermentation produces digestible proteins and peptides with antioxidant potential. Protein hydrolysate from chicken liver using lactic acid bacteria, *Pediococcus acidilactici* NCIM5368, was explored by Chakka et al. (2015). The hydrolysate evinced 96.14% DPPH radical scavenging activity and 95.02% superoxide anion scavenging activity (SASA).

## 3 Advanced Technological Approaches to Enhance Efficiency of Protein Hydrolysates

**FIGURE 2:**
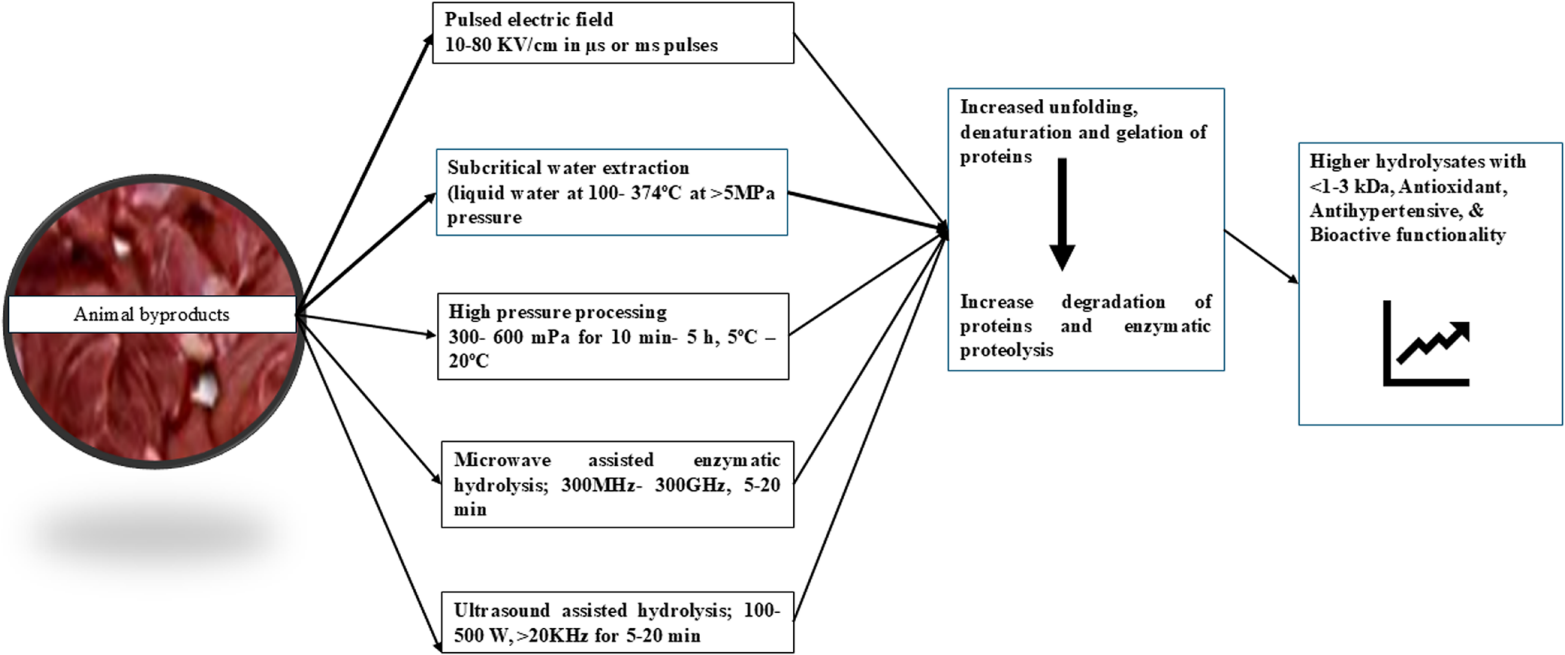
Novel technologies—Energy efficient, ecofriendly, improve yield and efficiency of enzymatic hydrolysis.

Nowadays, various modern techniques are being applied to enhance the functionality as well as yield of hydrolysates and/or peptides from animal‐origin proteins (Figure 2). For sustainable commercialization and industrial production, advanced technologies such as ultrasonication, pulsed electric field, high hydrostatic pressure, microwave, and subcritical water processing can be a promising approach. These high‐efficiency techniques could open up the protein structure, making unreachable sites available for the enzymes to act via different methodologies. Ultrasound‐assisted hydrolysis (20 kHz) of golden carp skin ( *Probarbus jullieni* ) by pepsin increased its yield from 79.27% to 94.88% over conventional methods (Ali et al. 2018). Pulsed ultrasound with single frequency countercurrent can be applied to meat processing coproducts for the generation of bioactive peptides. Chian et al. (2019) reported that the application of low‐intensity pulsed electric field on beef *longissimus thoracis* caused the weakening of the Z‐disk and I‐band junction, enhancing its accessibility to digestive enzymes without affecting the thermal denaturation temperatures of proteins. Pulsed electric field technology also induces partial unfolding of proteins and promotes bioactive compound extraction and recovery. The implementation of these modern technologies might open new avenues for the large‐scale production of bioactive peptides with higher efficiency and improved yield.

### 3.1 Ultrasonic‐Assisted Hydrolysis

It is the application of sound waves of frequency higher than 20 kHz. It can be applied in two modes: either low frequency and high power, that is, frequency higher than 16 kHz to 100 kHz and power from 10 to 1000 W/cm^2^ (high intensity/high power ultrasonics HIU) or high frequency and low power, where frequency ranges from 100 kHz to 10 MHz and power is kept below 1 W/cm^2^ (low intensity/low power ultrasonics) (Mehta et al. 2022). High‐intensity or high‐power ultrasonic processing has attracted interest in enhancing the efficiency of enzymolytic generation of bioactive peptides. Ultrasonic‐assisted hydrolysis with HIU works on the principle of acoustic cavitation.

Acoustic cavitation of the ultrasound creates mechanical and thermal effects, which are the ultimate processes responsible for modifying the physicochemical properties of a material. Acoustic cavitation generates extremely high local temperature and pressure variations causing micro jets and microturbulence, leading to accelerated reactions. The high mechanical shear forces disrupt hydrophobic interactions, hydrogen bonds, and complex structures (quaternary and/or tertiary) causing conformational changes. This resulted in the improvement of the physicochemical, functional, and antioxidant properties of protein hydrolysates (Ma et al. 2023). A higher degree of hydrolysis was observed in pork liver hydrolysates obtained by ultrasound‐assisted enzymatic hydrolysis (López‐Martínez et al. 2024). Vidal et al. (2018) reported alterations in the structural and functional properties of bovine collagen hydrolysates obtained by ultrasound‐assisted (88 w and 40 kHz frequency) hydrolysis by flavourzyme.

### 3.2 Pulsed Electric Field (PEF)‐Assisted Hydrolysis

Pulsed electric field processing has been widely explored for the preservation and processing of food products. It is a non‐thermal, short‐time processing technique that preserves food by the inactivation of microorganisms without affecting the quality characteristics such as flavor, color, and nutritional values. In the meat industry, PEF has also been used for improving the texture of meat by tenderization and curing (Kumar et al. 2020). The pulsed electric field works on the principle of electroporation, which involves the application of an electric field (0.1‐80 kV/cm) in short bursts (for nanoseconds to milliseconds at frequencies of 0.2–0.4 MHz). The product is placed in between two electrodes, and the electric field is applied for a short time. The intensity of the electric field applied produces varying permeabilization depending on the type of cells; that is, plant cells undergo reversible permeabilization on application of 0.1–1 kV/cm while at 0.5–3 kV/cm they undergo irreversible permeabilization, whereas microbial cells 15–70 kV/cm show irreversible permeabilization (Zhao et al. 2019). PEF technique has been effective in improving the efficiency of hydrolysis when applied as a pretreatment.

### 3.3 High Hydrostatic Pressure Processing

High hydrostatic pressure is an emerging technique that evolved as processing technology to destroy microorganisms, mainly for pasteurization of food to enhance their shelf life. HHP is a process of pascalization or cold pasteurization that works on Le Chatelier's principle. HHP involves the application of high isostatic pressure ranging from 100 MPa to 1000 MPa through a fluid medium containing the packaged product to be treated. This transmission of pressure is uniform, instant, and is not affected by the shape, size, or geometry of food. High pressure unfolds protein, specifically the secondary structure, as it disrupts the weak hydrophobic, ionic, and hydrogen bonds but does not affect the covalent bonds such as disulfide and peptidic (Marciniak et al. 2018). These changes open up the protein structure to make unavailable cleavage sites accessible to enzymes, as well as leave more charged groups on protein chains. Water percolates into the protein structure and increases the rate of mass transfer and protein enzyme contact (Bamdad et al. 2017). The efficiency of HHP‐assisted protein hydrolysis depends largely on the pressure, time, substrate (protein), and enzyme. Pressure ranges up to 100 MPa increase the susceptibility of proteins toward enzymes and enhance the short peptides content in the hydrolysate. High pressure from 100 to 150 MPa increases the accessibility of protein toward enzymes by dissociation of large protein molecules (Bamdad et al. 2017). Application of 400 MPa at 20°C for 15 min on hemoglobin samples improved its enzymatic hydrolysis by trypsin as well as also reduced the incubation time (Toldrà et al. 2011). The combination of HHP with enzymatic hydrolysis increases the efficiency and yield and significantly reduces the reaction time. HHP is an environment‐friendly technique that improves the efficiency of enzymatic hydrolysis.

### 3.4 Microwave‐Assisted Processing

Microwave heating depends very much on the dielectric properties of the medium, and during the hydrolysis of proteins, the process is speeded up by both the rotation of bipolar water molecules and protein ionic components. Conformational changes open up various inaccessible sites for enzyme action. Hall and Liceaga (2020) in their study reported a greater number of hydrophobic, positively charged, aromatic, and essential amino acids in microwave‐assisted hydrolyzed samples. The application of 300 W microwave power increased the hydrolysate content from 41% to 64% in the hydrolysis of sea cucumber ( *Acaudina molpadioides* ) collagen by pepsin (Jin et al. 2019). Microwave‐assisted hydrolysate also exhibited better DPPH scavenging capacity than conventional hydrolysate. The hydrolysate had a higher low molecular weight aromatic and/or acidic amino acids fraction. Pretreatment with microwave processing (800 W and 90°C for 5 min) increased the degree of hydrolysis and decreased the time of processing during the hydrolysis of fish frames (Ketnawa and Liceaga 2017).

The functional quality of hydrolysates is also improved by microwave processing. Nguyen et al. (2017) observed improved protein solubility, water absorption capacity and oil absorption capacity in hydrolysates obtained from Australian rock lobster. Similarly, in another study, Nguyen et al. (2017) applied 1200 W (20% power with 50% duty cycle at 50°C–55°C) for 5 min on alcalase hydrolysis of trout byproducts and observed a significantly higher degree of hydrolysis and protein solubility with higher emulsifying capacity and stability. Microwave‐assisted hydrolysates exhibited better FRAP and DPPH capacity than conventional hydrolysis.

### 3.5 Subcritical Water Processing

Subcritical water processing is a technique that involves heating water at a temperature between the boiling point of 100°C at atmospheric pressure and its critical point of 374°C at a pressure less than 22.06 MPa. Thus, water is maintained at its subcritical state at which it shows unique characteristics such as decreased surface tension, decreased viscosity, and altered dielectric properties with increased ionic groups leading to the formation of hydronium and hydroxide ions. This renders its unique potential to act as an acid or base catalyst which can hydrolyze proteins and amino acids without enzymes or acids. Such conditions become conducive for dissolving otherwise insoluble organic substances (Tkaczewska et al. 2021). Hydrolysis efficiency by subcritical water processing is increased as compared to conventional hydrolysis. Koh et al. (2019) reported a 62.7% degree of hydrolysis of bovine serum albumin, whereas the same by enzymatic hydrolysis is 26%–32%. Melgosa et al. (2020) utilized subcritical water at various temperatures (90°C, 140°C, 190°C, and 250°C) for the hydrolysis of sardine waste such as head, spin, and viscera obtained from a fish canning facility.

Defatting of sardine waste increased its protein recovery. The bioactivity of protein fractions showed variations based on the temperature of processing. The highest antioxidant activity, 3.56 mg extract/mg DPPH for sardine waste and 1.68 mg extract/mg DPPH for defatted sardine waste, was obtained at 250°C. Temperature and pressure variations change the yield and protein content in hydrolysates. Hydrolysis yield is increased by increasing temperature, but the protein content in hydrolysate is decreased at higher temperatures (Asaduzzaman and Chun 2015). The highest degree of hydrolysis of defatted bigeye tuna skin and collagen by subcritical hydrolysis was recorded at 250°C and 50 bar pressure, which decreased on increasing temperature to 250°C and was implicated to the decomposition of amino acids at higher temperature due to intemperate hydrolysis (Ahmed and Chun 2018). Jeong et al. (2021) attempted to generate free amino acids from blue mussel ( *Mytilus edulis* ) with subcritical water hydrolysis (temperature 120°C–240°C, pressure 3 MPa, time 30 min) and obtained the best hydrolysis efficiency of 90.65% at the highest temperature 240°C. Subcritical water hydrolysis of bovine serum albumin at temperatures 240°C, 260°C, 280°C, and 300°C revealed that the free amino acid content increased significantly with temperature rise, with the highest being 5.87 mmol/g protein at 280°C (Koh et al. 2019).

Asaduzzaman and Chun (2015) reported the highest yield of free amino acids in hydrolysate obtained from squid muscle hydrolyzed by subcritical water processing at temperatures varying from 160°C to 280°C, pressure from 6 to 66 bar for 3 min, at 250°C and the highest level of essential amino acids at 220°C, while the highest peptide concentration was achieved at 160°C. Authors suggested a reduction in peptides due to the degradation of complex amino acids at higher temperatures. The hydrolysate obtained at 220°C evinced the best anti‐oxidative potential with 98% ABTS antioxidant activity. Ahmed and Chun (2018) reported higher bioactivity of hydrolysates (tuna skin hydrolysate and collagen hydrolysate) at higher temperatures with the best antioxidant activity and antimicrobial activity in hydrolysates obtained at 280°C. Similarly, Jeong et al. (2021) obtained the highest antioxidant potential at 240°C during subcritical water hydrolysis of blue mussel (*Mytilus edulis*) but the best antihypertensive activity was observed in hydrolysate obtained at 180°C.

## 4 Assessing the Efficiency of Bioactive Peptides

### 4.1 Utilization of Bioactive Peptides in Meat Products

The importance of meat products in diet is indubitable. It is an important source of protein consisting of all essential amino acids, vitamins, and minerals. Similarly, fish and seafood are also excellent sources of proteins, minerals, vitamins, and essential fatty acids (Table 1). Globally, meat consumption increased by 58% over 20 years from 1998 to 2018, accounting for 360 million tons (OECD, Agricultural Outlook 2019). The major driving forces were the increased population, changing preferences, urbanization, and increasing disposable income. The processed meat products sector is a prominent industry. As per the Fortune Business Insight report, the global market size of processed meat products is USD 519.41 billion, which is expected to increase with a CAGR of 6.24% to reach USD 862.97 billion by 2027 (OECD, Agricultural Outlook 2019).

**TABLE 1:** Bioactive peptides generated by enzymes from animal and marine coproducts.

Products	Peptide source	Generation of peptides	Peptide attributes	Attributes	Findings	References
European dry‐fermented sausage	Intense degradation of titin and other muscle protein (pork)	Peptidase (endogenous and microbial)	Peptides varying from > 0.5 kDa)	Antioxidants (> 3 kDa); flavor (< 3 kDa); Antioxidant effect due to Glu, His, Ala, Phe, Val, Pro, Gly, Leu, and Ile	Peptide (< 3 kDa) improve flavor, DPPH radical‐scavenging activity (55%–73.7%) in the traditional dry‐fermented sausage	Gallego et al. (2017)
Squid	Gelatin hydrolysates from inner and outer tunic (Squid)	Seven commercial protease Protamex, Trypsin, Neutrase, Savinase, NS37005, Esperase, and Alcalase	Peptides with molecular weight 500–1400 Da	3–10‐fold increase in radical scavenging capacity of gelatin 25%–80% metal chelating capacity	FRAP increased twofold	Alemán et al. (2011)
Spaish dry‐cured ham	Muscle protein (pork)	Endogenous and microbial peptidase	SNAAC (MW 464.17 Da) most potent peptide	Good heat (up to 90°C) and salt stability (8%)	40% reduction in lipid oxidation in meat emulsion	Gallego et al. (2018)
	Titin (pork)	Endogenous muscle peptidase (exopeptidase and endopeptidase)	320 peptides	KDEAAKPKGPIKGVAKK, KKLRPGSGGEK, KNTDKWSECAR, and ISIDEGKVL identified at 9 months processing	Characteristics flavor and texture of traditional product	Gallego et al. (2015)
	Bone collagen (Femoral bone of pig)	Digestion and enzymatic proteolysis	Unique sequence of Gly‐Pro‐Hy	459 peptides derived from 57 proteins	Higher antioxidant capacity of cooked products than uncooked products	Gallego et al. (2017)
	Skeletal muscle (Biceps femoris of pig)		Water‐soluble peptide extract	73 types of peptides comprising 5–14 amino acid sequences	AAATP Most potent peptide	Escudero et al. (2013)
Dry‐cured ham and fermented sausage	Four target proteins glyceraldehyde‐3‐phosphate dehydrogenase, beta‐enolase, myozenin‐1, and troponin T (pork)	Muscle carboxypeptidase and microbial exo‐peptidase	di‐peptides as Ala‐Gln, Arg‐Gly, Asn‐Pro, lle–Leu, Ala‐Gly, Ser‐Gly, and Ser‐Gln; tri‐peptides like Ile‐Ile‐Pro, Arg‐Gly‐Ala, Gly‐Asn‐Pro, Gly‐Ala‐Gly, and Gly‐Pro‐Gly	Leu, Tyr, Lys, Ala, Gly, Glu, and Asp are important released amino acids in ham and Ala, Ser, Lys, Val, Tyr, and Arg in fermented sausage	Improving functional attributes of meat products	Mora, Gallego, Aristoy, et al. (2015)
Dry‐cured pork loin	Muscle (pork loins)	Probiotic action of lactic acid bacillus	Peptides with average molecular weight 13 kDa	Probiotic LAB do not have influence on the relative quantity of peptides	Improved water activity of 0.92–0.90 potential; DPPH‐75.27–79.57 and ABTS‐84.91–92.16	Kęska and Stadnik (2017)
*Sardinella aurita*	Myosin (Sardinelle)	Fermentation with *Bacillus subtilis* and *Bacillus amyloliquefaciens*	800 peptides, artificial synthesis of NVPVYEGY and ITALAPSTM, SLEAQAEKY, GTEDELDKY	DPPH‐ 30%–48%, β‐carotene bleaching IC 50‐0.21‐0.34	Fermented Sardinella is a good source of functional peptides that could be used in food as natural additives	Jemil et al. (2016)
Dry‐cured loins	Muscle (pork loin)	Fermentatio at 16°C and 70%–75% relative air humidity for 30 days, *Lactobacillus acidophilus* Bauer and *Bifidobacterium animalis* ssp. lactis BB126 log CFU/g of meat and glucose (6 g/kg of meat)	Peptides with MW below 3.5 kDa; highest antioxidant activity by SAGNPN, AAAAG, IHSGSVG, NVLVG, NAAKL, and GLAGA	Increased ORP (mV) to 382.70 and decreased water activity from 0.955 to 0.917 upon 9‐month storage at 4°C	A rapid increase in acidity increases proteolysis	Okoń et al. (2017)
Spanish Teruel, Italian Parma, and Belgian dry‐cured hams	Muscle proteins (mainly from myosin light chain, myosin heavy chain‐7, and myosin 4 [pig meat])	Muscle peptidase	9076 sequences of peptides	Deproteinized extract had high DPPH scavenging ability (50%–60%) and Fe2+ reducing power ability	Unique taste and flavor, natural source of added value to these products	Mora et al. (2016)
Fermented sausage	Actin, myosin light chain 1/3 (MLC 1/3), myosin regulatory light chain‐2 (MRLC‐2), myosin heavy chain (MHC), troponin T, and MRLC‐2	Exopeptidase from meat and Autochthonous starter culture of *Lactobacillus curvatus* CRL705 and *Staphylococcus vitulinus* GV318	33 peptides from myofibrillar fraction with 25 from actin	3211 bioactive Peptides with 327 sensory peptides and amino acids	Uniqueness of produced fermented sausages; as a quality markers	López et al. (2015)
Dry‐cured ham	Ubiquitin‐60S ribosomal protein	Ubiquitin proteasome system (UPS) consisting includes two ubiquitin‐activating enzymes (E1s), 40 ubiquitin conjugating enzymes (E2s), and > 500 ubiquitin ligases (E3s) occurs	68 peptides with 14 peptides identified at 9 months processing	Consecutive loss of amino acids, dipeptides, and tripeptides from both terminal sites	Peptides as safety and quality markers of processing	Mora, Gallego, Escudero, et al. (2015)
Harbin dry‐sausage	Pig muscle	Fermentation caused by *Pediococcus pentosaceus* (Pp), *Lactobacillus curvatus* (Lc), *Lactobacillus sakei* (Ls), and *Staphylococcus xylosus* (Sx)	—	Characteristics flavor and functionality of sausage due to generation of aldehydes, ketone, alcohol, and acids	Decreased water activity and moisture; increase in hardness and springiness	Hu et al. (2019)

The processed meat or fish products market can be a potential area for the consumption of bioactive peptides and can reduce the chemical load of products. Bioactive peptides are multifaceted entities that can be utilized in various forms. The various functional aspects of bioactive peptides that can be utilized by the meat and fish products sector have been explored and discussed in the following section.

### 4.2 Bioactive Peptides as Preservatives

Most of the processed meat or fish products are added with preservatives to enhance shelf life. Meat products during storage undergo several enzymatic changes which gradually decrease their acceptability. Rising consumer concerns about the carcinogenic in vivo activity of chemical preservatives have shifted researchers' interest toward clean labels and natural preservatives. Many natural preservatives are being explored widely for the preservation of meat products such as oregano and bay in meat breads (Umaraw et al. 2020). Recently, bioactive peptides and protein hydrolysates have shown a promising preservative effect on various food items such as pork loaves (Verma, Chatli, Kumar, et al. 2022). The structure, sequence, helicity, charge, and hydrophilicity or hydrophobicity define the activity of bioactive peptides.

#### 4.2.1 Antioxidant

The antioxidant activity of bioactive peptides is attributed to their electron or hydrogen donors, radical stabilization, chelation of metal ions, and inhibition of penetration or diffusion of lipid oxidation initiators by forming a physical barrier around lipid droplets due to their increased charge and reduced size, making them easily distributed in the aqueous phase and absorbed at the oil–water interface. The presence of higher amounts of hydrophobic amino acids and residues of Histidine, Proline, Methionine, Cysteine, Tyrosine, Tryptophan, and Phenylalanine amino acids resulted in increased interaction with lipid molecules and contributed to scavenging radicals by donating protons (Lorenzo et al. 2018; Gallego et al. 2017). Animal/fishery byproduct hydrolysates and peptides have also shown interesting and promising antioxidant potential that poses a cogent alternative to chemical preservatives.

Proteins, protein hydrolysates, individual peptides, and amino acids have been shown to possess significant antioxidant activities. However, in many cases, peptide fractions or protein hydrolysates display greater antioxidant activity than intact proteins or free amino acid mixtures, suggesting that peptides play a major role in the antioxidant action of proteins. More recently, individual peptides responsible for the antioxidant activity of protein or protein hydrolysates have been separated and identified. Protein digests have varied antioxidant activities depending on the peptide structure, that is, the size of the peptides and their amino acid sequences, which are influenced by the source of protein and conditions of the hydrolysis process involved. There appears to be a relationship between the antioxidant properties of peptides and the presence of aromatic, imidazole, sulfur‐containing amino, and imino acids. The antioxidant mechanism (structure–activity relationship) of peptides is not yet fully understood. The radical scavenging of aromatic amino acid‐containing peptides is likely due to the hydrogen atom donor activity of the phenolic and indolic groups and the higher stability of the phenoxyl and indolyl radicals than that of the simple peroxyl radical.

Structural properties, chemical structure, temperature, substrate characteristics, pro‐oxidants presence, and the physical state of the system determine the efficiency of antioxidants. Chemical assays such as radical/reactive oxygen species (ROS) scavenging methods (Oxygen radical absorbance capacity (ORAC) assay, chemiluminescence, 2,2‐di (4‐tert‐octylphenyl)‐1‐picrylhydrazyl (DPPH) radical scavenging assay, 2,20‐Azinobis‐(3‐ethylbenzothiazoline‐6‐sulfonic acid (ABTS), Trolox Equivalent Antioxidant Capacity (TEAC), and redox potential assay (Ferric Reducing Antioxidant Power [FRAP] assay, CUPRAC, Ag + reducing, Au 3+ reducing, CERAC, CHROMAC) have been developed to assess the antioxidant potential. Radical scavenging assays measure hydrogen atom transfer or single electron transfer from the antioxidant to the free radicals generated in the system, indicating the intrinsic antioxidant potential of the compound unaffected by the environment. Based on the chemical reactions, these assays are classified as hydrogen atom transfer (HAT) reaction‐based assays and single electron transfer (ET) reaction‐based assays. These methods are widely used for the initial screening of novel antioxidants. Hydrolysates and bioactive peptides obtained from animal and fish byproducts have been evaluated based on these chemical assays and have reported promising results.

Wang et al. (2013) relied on DPPH radical scavenging assay as an easy, reliable, and quick method to assess the free radical scavenging or hydrogen donor activity of blue mussel ( *Mytilus edulis* ) protein hydrolysate. Four proteases namely alcalase, pepsin, papain, and neutrase were used for hydrolysis (10 mg/ml) and reported time‐dependent antioxidant efficiency of hydrolysates. As the time of hydrolysis increased the antioxidant activity increased, reached an optimum, and then started decreasing for pepsin, papain, and neutrase enzymes, of which neutrase at 3 h hydrolysis evinced the highest 28.8% ± 1.79% DPPH radical scavenging activity. This hydrolysate was further fractioned based on molecular weight (< 3, 3–10 kDa and > 10 kDa). The fraction with MW < 3 kDa exhibited superior DPPH radical scavenging activity of 35.7% ± 2.01%. The higher radical scavenging activity of this fraction was attributed to its lower molecular weight. Centenaro et al. (2014) attempted to characterize Argentine croaker ( *Umbrina canosai* ) fish protein hydrolysate and evaluate the antioxidant potential of its fractions. Compositional analysis of hydrolysate produced by flavourzyme revealed presence of 50.9% peptides with molecular weight less than 3 kDa. Differentiation of hydrolysate on molecular weight MW > 1, 0.5–1 and < 0.5 kDa was done by ultrafiltration membranes and were analyzed for their antioxidant potential by sequestration ability of the hydroxyl radical (OH·), 2,2‐diphenyl‐1‐picryl‐hydrazyl (DPPH) radical scavenging, ABTS free radical scavenging and reducing power.

Hydrolysates obtained from the digestion of porcine liver with alcalase, trypsin, and papain were evaluated by Verma et al. (2019b) for their antioxidant potential. The hydrolysate obtained by the action of trypsin recorded the highest antioxidant potential as evinced through its ABTS percent inhibition (86.83%), DPPH inhibition (56.67%), and FRAP values (14.38 mM equivalents to ferrous sulfate). The hydrolysates obtained were grouped into their molecular weight range of < 1 kDa, 1–5 kDa, 5–10 kDa, and > 10 kDa to identify the most effective anti‐oxidative fraction. Fraction 5–10 kDa evinced the best antioxidant values, but these were lower than the values obtained from their respective whole hydrolysate. This better performance of whole hydrolysate than its fractions was attributed to the synergistic and/or additive effect of all fractions in the whole hydrolysate.

López‐Pedrouso et al. (2020) identified the most abundant antioxidant peptides obtained from the hydrolysis of the porcine liver with alcalase, bromelain, flavourzyme, and papain separately. Bromelain‐induced hydrolysis of the liver produced the GLNQALVDLHALGSAR peptide, which evinced the highest antioxidant potential with an ORAC value of 0.743 mg Trolox Equivalent (TE)/g while others such as ALFQDVQKPSQDEWGK obtained from flavourzyme and LSGPQAGLGEYLFER produced by the papain enzyme showed ORAC values of 0.605 and 0.682 (TE)/g, respectively. Meanwhile, LGEHNIDVLEGNEQFINAAK, a trypsinogen origin peptide, was also produced by the papain enzyme and reported propitious antioxidant potential with an ORAC value of 0.619 (TE)/g, 0.789 ABTS, and 0.592 FRAP values.

Protein derived from blue mussel ( *Mytilus edulis* ) was hydrolyzed using four kinds of proteases (pepsin, papain, neutrase, and alcalase), and the neutrase hydrolysate (BNH) obtained by 3‐h hydrolysis exhibited the highest 2,2‐diphenyl‐1‐picrylhydrazyl (DPPH) radical scavenging activity compared to other hydrolysates. By using ultrafiltration, gel filtration chromatography, and reversed‐phase high‐performance liquid chromatography (RP‐HPLC), a novel antioxidant peptide (BNH‐P7) was isolated from BNH, and its amino acid sequence was identified as YPPAK (Tyr‐Pro‐Pro‐Ala‐Lys) with a molecular weight of 574 Da. BNH‐P7 exhibited good scavenging activity on DPPH radical, hydroxyl radical, and superoxide anion radical with EC50 of 2.62, 0.228, and 0.072 mg/mL, respectively. BNH‐P7 was also effective against lipid peroxidation in a linoleic acid model system. The high activity of BNH‐P7 was due to the small size and the presence of antioxidant and hydrophobic amino acid residues (Tyr and Pro) within its sequence (Wang et al. 2013).

Centenaro et al. (2014) prepared Argentine croaker ( *Umbrina canosai* ) fish hydrolysate with 25.9° of hydrolysis (DH) by using flavourzyme (FHF) and α‐Chymotrypsin enzymes, leading to 60.8% and 50.9% peptides with a molecular weight less than 3 kDa. F1000 fractions obtained from FHF hydrolysates exhibited higher antioxidant potential owing to a higher concentration of amino acids having hydrophobic and sulfuric residues. Its 40 mg/mL incorporation in ground beef homogenates was able to retard lipid oxidation to a major extent (93%). The addition of Rainbow trout byproduct hydrolysate washed with distilled water BPW‐FPI (1–3 g/100 g) controlled the oxidation of fish emulsion in a dose‐dependent pattern during storage for 6 days at refrigeration (Nikoo, Benjakul, Yasemi, et al. 2019).

Bioactive peptides are capable of chelating metal ions, particularly iron and copper, thereby increasing the oxidative stability of meat products by retarding lipid oxidation‐caused food deterioration, off odor, and oxidative stress‐mediated cellular injury. This activity is attributed to the imidazole ring of histidine and peptide size. Mineral‐binding bioactive peptides have a unique property due to their binding with metal; it helps in the in vivo absorption of metals, such as calcium, iron, zinc, copper, and other trace elements, thus enhancing their bioavailability.

#### 4.2.2 Antimicrobial

**FIGURE 3:**
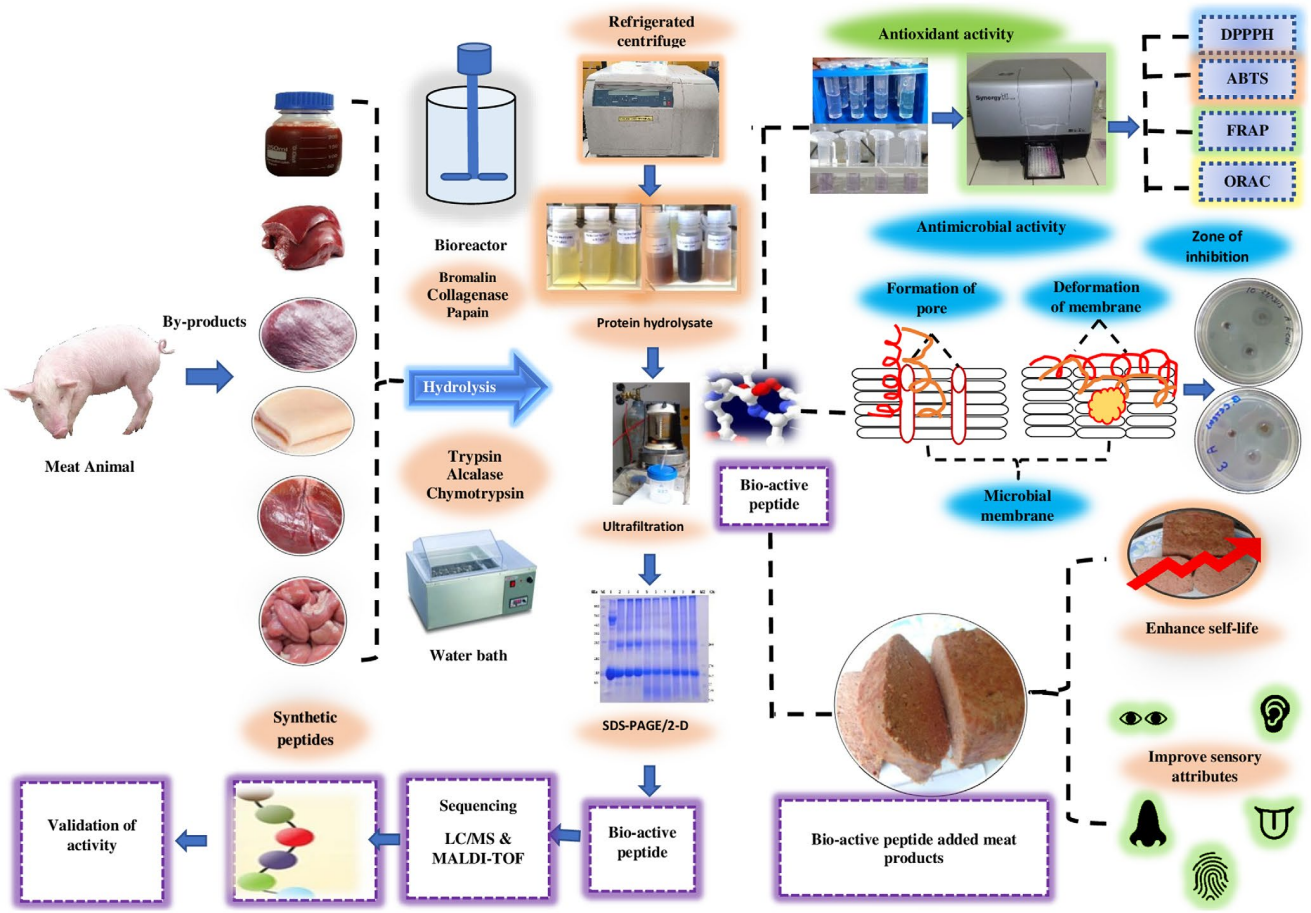
Production of bioactive peptides from meat byproducts and their characterization with functional activities.

The antimicrobial activity of peptides varies with the net charge, helicity degree, hydrophobicity, and amino acid sequences. In general, these peptides have lower antimicrobial potential than synthetic antimicrobials, but their wide spectrum activity and fast bactericidal effect could be very beneficial for the food industry (Figure 3).

The potent host‐defense substances attribute antimicrobial activity against a range of pathogens of short bioactive peptides containing 10–50 amino acids exhibiting cationic and hydrophobic properties. The antimicrobial property of bioactive peptides is widely attributed to the carpet model of membrane disruption comprising the accumulation of these peptides on membrane surfaces by electrostatic interactions (Fernandez et al. 2012) and upon reaching a threshold or critical level leads to increased membrane fluidity and permeability. Some peptides, especially those having a cationic charge, can enter the cell by crossing the lipid bilayer and have antimicrobial effects by targeting intracellular components such as the inhibition of enzymes, phospho‐proteins, or nucleic acid synthesis. A novel antimicrobial peptide obtained from α‐chain bovine hemoglobin, VNFKLLSHSLLVTLASHL, exhibited an antimicrobial effect against *E. coli* , *S. aureus* , and *Candida albicans* (Hu et al. 2011).

There are various animal protein hydrolysates having potential antimicrobial activity against a range of pathogens that have been identified, and their utilization as natural antimicrobials or antibiotic compounds is being explored for treating animal diseases. Bioactive peptides exerting antimicrobial effects have been identified in bovine hemoglobin hydrolysates (Catiau et al. 2011; Lafarga and Hayes 2014), in seafood skin hydrolysates (Anal et al. 2013; Guillén et al. 2010), and barbell muscle hydrolysates (Sila et al. 2013).

#### 4.2.3 Antifungal

Peptides such as histatins or bacterial lipopeptides exhibit antifungal properties. Similar to antimicrobial peptides, these peptides are small in size and have cationic and hydrophobic residues. These antifungal peptides have linear structures forming α‐helix in a hydrophobic environment, β‐sheet containing several bonds, CSαβ peptides of helix (α or 310) and β‐sheets formed by disulfide bonds; α‐hairpin or SS bond‐containing loop; and linear peptides having specific residues (Wang et al. 2013).

The peptide net charge is controlled intrinsically by the presence of polar, positively (Arg, Lys, and His) or negatively (Glu, Gln, and Asp) charged residues. A positive net charge (cationicity) is generally associated with a strong interaction with the negatively charged cell membrane of microbes. Hydrophobicity, or hydrophobic content, is based on the proportion of hydrophobic residues (Val, Ile, Trp, Phe, Leu, Cys, Met, and Ala) in the peptide. A minimal hydrophobicity is required for the penetration and binding of peptides into the bilayer of the cell membrane.

The comparison of different peptide sequences and structures has highlighted the key role of several parameters in antifungal potency, such as the (cationicity) net charge, the hydrophobicity, and the distribution of the residues and associated structure, length, and amphipathicity. These properties can be modified in the design of optimized peptides (Figure 3), including shorter sequences, higher stability to heat treatment, and resistance in high salt or serum concentrations.

## 5 Bioactive Peptides in Improving Functionality in Meat Products

In addition to exerting biological activities, BP derived from animal byproducts has a major role in improving the functionality and quality of meat and meat products such as improving oxidative stability and improving quality characteristics such as color, texture, water activity, and sensory attributes (Table 2). The controlled enzymatic hydrolysis has been used to improve protein functionality and solubility by cleaving peptide bonds, leading to an increased concentration of free amino acids and carboxyl groups. Hydrolysis destroys the protein tertiary structure, decreases molecular weight, and consequently, alters the functional properties of proteins.

**TABLE 2:** Bioactive peptides and their various functional uses in meat and meat products.

Product	Hydrolysate/peptide	Enzyme used	Mode/level of incorporation	Effect	Reference
Fish tofu	Shrimp hydrolysate	Farmed giant catfish viscera protease (G) (8 U/g protein) at 50°C, pH 8 for 3 h	0.05%, 1.00%, and 2.00% (w/w)	Controlled lipid oxidation, lower thiobarbituric acid‐reactive substances (TBARS), and decreased microbial spoilage	Ketnawa et al. (2016)
Turkey meat sausage	Goby (*Zosterissessor ophiocephalus*) fish protein hydrolysates	Gray triggerfish proteases (GPH‐TF)	0.01%, 0.02%, 0.04%, 0.1%, and 0.2% (w/w) of GPH‐TF powder	Inhibited the TBARS formation during storage	Nasri et al. (2013)
Soy‐based extruded meat alternatives	Maillard‐reacted beef bone hydrolysate (MRP)	Flavourzyme	0%, 10%, 20%, 30%, and 40% wet weight	Appearance, meaty aroma, meaty taste, and overall acceptability	Chiang et al. (2020)
Dressed sardine during ice storage	Tuna ( *Euthynnus affinis* ) protein hydrolysate	Papain enzyme (0.5% w/w)	Dip treatment in solution	Reduced oxidation in ice stored dressed sardine	Parvathy et al. (2018)
Imitation fish paste (IFP) made from surimi containing Alaska Pollack, spent laying hens	Mechanically deboned chicken meat (MDCM) hydrolysates	1% Bromelain	0%, 0.4%, and 0.8%	Improved gel characteristics, inhibitory Angiotensin I‐converting enzyme (ACE) activity, and increased free radical scavenging activity	Jin et al. (2014)
Minced meat (Beef meat patties uncooked)	European eel ( *A. anguilla* ) byproducts hydrolysates	Savinase Protamex	0.5% and 1% w/w	Reduced the lipid oxidation and inhibited the microbial growth during chilled storage for 11 days	Bougatef et al. (2020)
Mayonnaise	Byproduct from tuna canning industry—red meat of yellowfin tuna ( *Thunnus albacares* )	Papain	Replacing 15% egg yolk by 5 g, 7.5 g and 10 g of	Higher emulsion quality, better oxidative and physicochemical stability	Unnikrishnan et al. (2020)
Fish mince	Rainbow trout ( *Oncorhynchus mykiss* ) mixed processing byproduct	Alcalase	8% hydrolysate powder, 4% hydrolysate powder +4%	Good antifreeze activity, decreased the loss of total sulfhydryl groups, protein solubility and protein carbonyl formation after six freeze–thaw cycles, and potential use as additive in frozen fish mince	Nikoo, Benjakul, Gavlighi, et al. (2019)
Seal meat	Seal meat hydrolysates	—	—	Good antioxidant activity Can be used as preservatives to control oxidation in food	Zhang et al. (2022)
Pork meat balls	Cracklings hydrolysates	Alcalase L 2.4; 4.75 M HCl	2% in formulation of meatballs and stored for 7 days under refrigerated	Better inhibition of cholesterol oxidation than BHT, but lower antioxidant potential than BHT	Flaczyk et al. (2006)
Pork patties	Plasma protein (PP) hydrolysates	Alcalase	2% in the formulation	Delayed lipid oxidation	Seo et al. (2016)
In vitro	Blood hydrolysate	Alcalase, trypsin, and papain	Different molecular weight (< 5, 5–10, and > 10 kDa)	DPPH, ABTS, and FRAP activity zone of inhibition against *Escherichia coli* , *Bacillus cereus* , *Staphylococcus aureus* , and *Listeria monocytogenes*	Verma, Chatli, Mehta, et al. (2017)

The blood, also known as liquid protein, is the first animal byproduct obtained during slaughter. A huge amount of blood is generated during slaughtering, and its proper disposal is very critical due to its high pollutant characteristics. The blood protein hydrolysates could be potentially used for increasing the final nutritional value of foods and enhancing functional properties like water binding and emulsifying capacity (Verma, Chatli, et al. 2018).

### 5.1 Water Binding/Holding Capacity

Product yield is an important aspect of product development which is influenced by the water‐holding capacity (WHC) of the product. WHC affects the elasticity, mechanical strength, viscosity, gelation, and surface properties. The water‐holding capacity of peptides or hydrolysates is governed by the pH, enzyme, concentration, and molecular weight. Among hydrolysates, purified hydrolysates have reported better water‐holding capacity than crude hydrolysates (Nuñez et al. 2020). Proteins with a higher degree of hydrolysis produce hydrolysates with better water‐holding capacity as they expose the active polar groups in proteins.

The water‐holding capacity of meat, meat products, or emulsion is improved by increasing its pH above the isoelectric point. The addition of hydrolysates tends to increase the pH of the meat model system and improve its water‐holding capacity (Glorieux et al. 2017). Low molecular weight peptides are more competent in improving the water‐holding capacity, as they have higher hydrophilic amino acid residues. Nuñez et al. (2020) compared the efficiency of bovine skin gelatin hydrolysate with sodium tripolyphosphate (STPP) for the effect on the water‐holding capacity of thermally processed chicken meat. It was observed that the incorporation of hydrolysate at 0%–5% w/w improved the water‐holding capacity and cooked product yield in a dose‐dependent manner, which was concomitant with that of STPP. Although higher WHC is associated with high hydrophilic groups containing hydrolysates, fractionation of hydrolysate on molecular weight and hydrophilicity does not increase the WHC above that of the whole hydrolysate. This has been attributed to the synergistic effect of all fractions in the whole hydrolysate (Nuñez et al. 2021).

### 5.2 Bioactive Peptides and Meat Emulsion Properties

Controlled enzymatic hydrolysis might be used to generate hydrolysate or peptides which improve the functional properties of food. Hydrolysis causes the cleavage of peptidic bonds to convert tertiary and secondary structures into smaller fragments with increased free amino and carboxyl groups. These free groups are responsible for altered functional properties like solubility. However, unrestrained hydrolysis might cause excessive degradation resulting in loss of functional properties. Liu et al. (2022) observed the effect of hydrolysis by neutral protease (EHN), trypsin (EHT), and alkaline protease (EHA) on hen eggs' high‐density lipoprotein (HDL) and observed that the solubility of hydrolysate obtained by alkaline protease increased from 7.69% to 27.54% when hydrolyzed for 1.5 h. Leni et al. (2020) generated insect protein hydrolysate from lesser mealworm ( *Alphitobius diaperinus* ) with the protease. This hydrolysate evinced high solubility with 95% solubility at pH of 3, 5, and 7. It also showed a five times higher oil holding capacity (6.7 ± 0.6 g oil per g) than casein and egg white. The protein hydrolysate forms a protective layer around fat molecules and increases its oil holding capacity (Verma, Chatli, Mehta, et al. 2022). Peptides obtained by hydrolysis of myofibrillar proteins have proportionately higher polar residues that tend to form hydrogen bonds in the presence of water and thus increase solubility.

Molecular weight plays a vital role in the functional properties of hydrolysates. Shaik et al. (2021) observed the functional properties of various fractions viz. fraction with molecular weight < 10 kDa, fraction with MW < 5 kDa, and fraction with < 3 kDa obtained by hydrolysis of silver catfish muscle proteins. The solubility of the fraction < 3 kDa was the highest, but the emulsion stability index of fraction < 10 kDa was the highest. Small‐size peptides are highly hydrophilic and thus have higher solubility in aqueous medium. The emulsion stability of lower weight fractions is lower than the high molecular weight fraction due to its frequent migration into the interface and because of the unstable balance between the hydrophilic and hydrophobic groups. However, the emulsion activity index of lower weight fractions is better than that of the higher weight fraction. Razali et al. (2015) reported that hydrolysates with smaller molecular weight evince higher emulsifying activity index (EAI) and better water binding efficiency than the higher molecular weight fractions. Among fractions, the lower molecular weight peptides migrate rapidly in the oil and water interface, exhibiting better EAI, whereas higher molecular weight fractions have better oil binding and show higher emulsifying stability index.

In the meat model system, incorporation of hydrolysate in pork loaves improved the emulsion stability of the pork emulsion, which was attributed to the presence of both hydrophilic and hydrophobic groups that interacted with both water and fat molecules and prevented their loss during cooking (Verma, Chatli, Kumar, et al. 2018; Verma et al. 2019c). Hydrolysates also participate in fat membrane formation, which prevents aggregation of lipid molecules in emulsion and improves the emulsion stability (Verma, Chatli, Mehta, et al. 2018). Thus, controlled enzymatic hydrolysis can be used to produce peptides with desirable molecular weight.

Intarasirisawat et al. (2014) observed higher emulsifying activity in sausages containing higher levels of skipjack roe protein hydrolysate (SRPH) that was attributed to SRPH's emulsifying properties. The authors proposed that SRPH and myofibrillar proteins migrated to the interfacial surface and stabilized the emulsion.

Verma, Chatli, Mehta, et al. (2022) reported that the emulsion stability of liver protein hydrolysate incorporated samples was higher than that of the control. It decreased significantly (*p* < 0.05) among all groups during storage. However, ES was better maintained in the treatment group than in the control. Protein hydrolysate might have formed a protective film around lipid molecules in treated samples. The presence of hydrophilic and hydrophobic peptides in hydrolysate may have also interacted with water and fat molecules in the meat matrix system, which prevented the leaching of lipid and water molecules during cooking (Verma et al. 2021). The decrease in emulsion stability with storage might be due to microbial breakdown of protein, enzymatic, and non‐enzymatic oxidation of lipid molecules. Verma, Chatli, Kumar and Mehta (2019) also reported that the addition of meat hydrolysates in meat batter decreased the surface tension and acted as an emulsifier; therefore, it reduced the formation of fat pockets, decreased the loss of water and fat during cooking, as well as increased the quality of products.

### 5.3 Foaming Capacity

The measurement of interfacial area created during whipping gives the foaming capacity of a protein, while foaming stability is the measure of time that elapses to lose either 50% of foam volume or liquid. Unfolding of proteins by any means discloses the hydrophobic sites, which readily adsorb to the air–water interface. Protein hydrolysis causes cleavage of peptidic bonds, unraveling the protein structure to expose the hydrophobic groups. Thus, proteins competent in unfolding, migration, and rearrangement at the interface tend to have good foaming capacity. The foaming properties of protein depend on various factors such as pH, net charge, solubility, isoelectric pH, and molecular weight of peptides. The effect of enzymes used for hydrolysis has also been reported to affect the foaming properties (Klompong et al. 2007).

Peptides with available hydrophobic and hydrophilic sites possess the ability to stabilize the foams by inculcating themselves within the air and water interface. The improved solubility also contributes to improving foaming ability. Tang et al. (2021) studied the effect of peptides obtained from soy proteins, corn proteins, whey proteins, and fish skin proteins on the foaming attributes of egg white powder. The fish skin peptides improved the foaming properties of egg white powder. The foaming properties of fish protein might have increased the flexibility of proteins, exposed more hydrophobic residues, or might have decreased the surface tension. Thus, peptides prevented the coalescence of air bubbles formed and rendered stability to the foam. The efficiency of fish protein peptides was independent of the solubility. Peptides, due to their flexible structure, rearrange themselves in the air–water interface and during the agitation process, and undergo conformational changes or rearrangement to stabilize the foam. The peptides also increased the flexibility of egg white proteins by altering their secondary structure (Tang et al. 2021).

The effect of the molecular weight of peptides on the foaming capacity was studied by Shaik et al. (2021) with silver catfish muscle protein hydrolysate. The hydrolysate obtained from silver catfish was fractioned into fractions with molecular weights < 10, < 5, and < 3 kDa. The fraction with molecular weight < 10 kDa observed the highest foaming capacity of 30.39%, which was attributed to the polypeptides that formed a stable film around the air bubbles (Chi et al. 2014). The protein–protein interaction, protein structure, and protein flexibility govern the stability of foam with peptides or hydrolysates. Low molecular weight peptides might form better foam, but the stability of such foams is low due to rapid migration.

### 5.4 Textural Attributes

Meat products incorporated with protein hydrolysate have been observed to improve the textural attributes of meat products. The incorporation of protein hydrolysates leads to a weaker and more deformable gel structure, probably due to decreased binding among proteins. The addition of skiproe hydrolysate at all levels increased the hardness, chewiness, and resilience of fish emulsion‐type sausages (*p* < 0.05) but the springiness was not much affected (Intarasirisawat et al. 2014). Similarly, Cavalheiro et al. (2014) reported lower hardness values in mortadella sausage prepared with the incorporation of mechanically deboned chicken meat hydrolysate at 10%, 20%, and 30%. The effect of incorporation was inversely related to the level of incorporation. All the texture parameters, such as springiness, gumminess, cohesiveness, and chewiness, decreased with increasing levels of hydrolysate.

Contrarily, Zakaria and Sarbon (2018) observed increased hardness, cohesiveness, and springiness in fish emulsion sausages incorporated with hydrolysate obtained from fish (snakehead) proteins. Meanwhile, Verma, Chatli, Mehta, et al. (2022) reported nonsignificant changes in texture parameters such as hardness, springiness, stringiness, cohesiveness, chewiness, gumminess, and resilience of pork loaves developed by incorporation of liver and blood hydrolysates independently.

### 5.5 Color Profile

Sight or visual appeal is the first sensory perception of food received, and the color of meat products is the chief cue influencing the purchasing attitude of consumers (Umaraw and Verma 2016). Color in fresh meat is the major determinant of purchasing behavior. Instrumental color evaluation depends on assessing parameters such as the lightness/brightness (L*), redness (a*), and yellowness (b*) values that describe colors perceived by the human eye. The measurement of L* ranges from 0 to 100, which corresponds to black to white; the a* value expressed as redness is measured from green (−) to red (+) and the yellowness value b* ranges from blue (−) to yellow (+). This gives a three‐dimensional color space, where a* represents the *X*‐axis, b* value is denoted as the *Y*‐axis, and the *Z*‐axis represents L* values. The effect of hydrolysates on color changes in meat products has also been explored (Sun and Xiong 2015; Jin et al. 2015; El‐Saadony et al. 2021).

Hydrolysates have shown color stabilization potential when incorporated in meat product formulation. Similarly, a lowering in lightness value was also observed by Verma, Chatli, et al. (2018) in pork emulsions added with blood hydrolysate. The lower L* value was attributed to the inherent red color of the blood hydrolysate and discoloration during storage was ascribed to the meat pigment interaction with the products of lipid oxidation as well as to the moisture loss. During storage, the color of the emulsion was better maintained in hydrolysate‐containing emulsion. The color changes and the better‐maintained color in meat products during storage were implicated in the slower oxidative changes in products incorporated with hydrolysates. Hydrolysates have antioxidant potential which prevents color fading also. Abu‐Salem et al. (2014) correlated the color stabilizing effect of protein hydrolysate in beef burgers to retarded oxidation of metmyoglobin. Similarly, Verma, Chatli, Kumar and Mehta (2019) observed lower color deterioration in meat emulsion incorporated with porcine liver hydrolysate. Whereas, Zakaria and Sarbon (2018) did not observe any significant changes in L*, a*, and b*values of fish, shortfin scad emulsion sausage by incorporation of hydrolysate obtained from *Channa striata* protein. Although, the addition of hydrolysate retarded lipid oxidation in sausages containing 3% hydrolysate for 12 days of storage.

### 5.6 Sensory Quality

Incorporation of bioactive peptides of byproduct origin in meat products should surpass the acceptability threshold at the research as well as the consumer level (Verma, Umaraw, et al. 2019). Most of the studies related to the incorporation of peptides in meat products or food items have comprehended this concern and have contemplated its effect on sensory attributes. Sensory attributes such as color, appearance, taste, flavor, texture, and juiciness in meat products have been analyzed to evaluate changes in quality during product development. Jin et al. (2021) did not observe significant variations in the sensory attributes except the color of control and porcine plasma hydrolysate incorporated emulsion sausages stored for 4 weeks at cold storage. The treatments showed a darker color than control which might be attributed to the darker color of hydrolysate as the dark color increased in a dose‐dependent manner. Whereas, Verma, Chatli, Kumar et al. (2022) received higher color and juiciness scores for blood hydrolysate (600 mg/g) added pork loaves and also for liver hydrolysate (600 mg/g) incorporated pork loaves that were attributed to the color development due to the interaction of peptides and sugar generating Maillard reaction products. The appearance and flavor scores were also rated higher in treatments.

Meat and meat products have characteristic sensory attributes due to the presence of many water‐soluble compounds such as peptides, inorganic salts, organic acids, amino acids, sugars, and ATP breakdown products. Thus, products developed by incorporating meat hydrolysates or meat coproducts hydrolysates have better flavor due to the inherent presence of umami peptides such as aspartate and glutamate or flavor peptides. Flavor peptides are oligopeptides having a molecular weight < 3 kDa and are produced during the synthesis of amino acids or by enzymatic hydrolysis. Some flavor‐enhancing, umami peptides obtained during enzymatic hydrolysis are Gly‐Asp, Ala‐Glu, Gly‐Asp‐Gly, Val‐Asp‐Val, Asp‐Leu and Val‐Glu‐Leu, Glu‐Glu, Glu‐Val, Ala‐Asp‐Glu, Ala‐Glu‐Asp, Asp‐Glu‐Glu, and Ser‐Pro‐Glu. Depending on the size, these oligopeptides impart unique tastes such as sweet (Gly‐Gly, Gly‐Gly‐Gly, Gly‐Gly‐Pro), sour (Asp‐Trp‐Asp‐Ser, Ala‐Lys‐Ser‐Pro‐Lys‐Lys‐Pro, Gly‐Gln‐Ala‐Arg), salty (1‐Orn‐Gly‐Gly‐Gly‐Orn‐Gly‐Gly‐Orn‐1‐Orn, 2‐Orn‐Gly) bitter (hydrophobic peptides), umami (Asp, Glu), and sixth taste kokumi (γ‐glutamyl peptides). Kokumi taste imparts a sense of mouthfulness and thickness, inducing a lasting pleasant taste.

Zhan et al. (2013) analyzed the changes in mutton process flavor on hydrolysis of sheep bone by chromatography and descriptive sensory evaluation. As the degree of hydrolysis increased, the free amino acids increased from 3.7784 mg/g to 32.7527 mg/g dw. Free amino acids are associated with characteristic cooked meat flavor and meaty and roasty aroma. The amino acids identified were arginine, aspartic, glycine, leucine, valine, alanine, phenylalanine, threonine, tyrosine, glutamic, and cysteine, which were similar to those found in goat meat.

Peptides containing hydrophobic groups tend to have a bitter taste. However, the intensity of bitterness varies with length, amino acid order, and structure. The bitterness of peptides having a molecular weight < 6 kDa can be predicted by the Q value. Peptides with a Q value higher than 1400 cal/mol impart a bitter taste, while those having a value below 1300 cal/mol are non‐bitter. It is difficult to predict the *Q* value of a mixture of peptides or hydrolysate, but the *Q* value of proteins can also be used as a measure of the hydrophobicity of peptides. Similarly, the sequence of amino acids and spatial arrangement also play a critical role in imparting bitterness (Fu et al. 2018). It has been reported that a high degree of hydrolysis releases the amino acids from bitter peptides, which enhances umami peptides in hydrolysates (Rhyu and Kim 2011).

Zhang, Tong, et al. (2019), Zhang, Cao, et al. (2019), Zhang, Ma, (2019) identified three peptides WVNEEDHL (Octa‐), NSLEGEFKG (nona‐), and KDLFDPVIQD (deca) with molecular weights ranging from 1 to 5 kDa, having umami and kokumi enhancing effects from chicken protein hydrolysate by ultrafiltration chromatography and sensory evaluation. These peptides appreciably increased the meat flavor, umami, and kokumi taste and can potentially be used as chicken flavoring in the food industry.

Meat and meat coproducts are rich in proteins containing glutamic acid, and to harness the kokumi effect of γ‐glutamyl peptides, efforts are being made. Li et al. (2020) contemplated the effect of γ‐glutamyl transpeptidase in enhancing the kokumi sensation of pig hemoglobin and pork meat hydrolysates. Quantification and sensory evaluation revealed that γ‐glutamylation appreciably increased the formation of γ‐glutamyl dipeptides and free amino acids with intense kokumi sensation. It was also reported that the kokumi sensation of protein hydrolysates could be enhanced by the external addition of glutamine (10–20 mM) followed by γ‐glutamylation.

Hydrolysates from meat coproducts with high functionality can also be used as flavorants or natural flavoring agents by γ‐glutamylation. It might be a potential method of increasing the sensory acceptability of hydrolysates in products.

## 6 As Component of Packaging System

**FIGURE 4:**
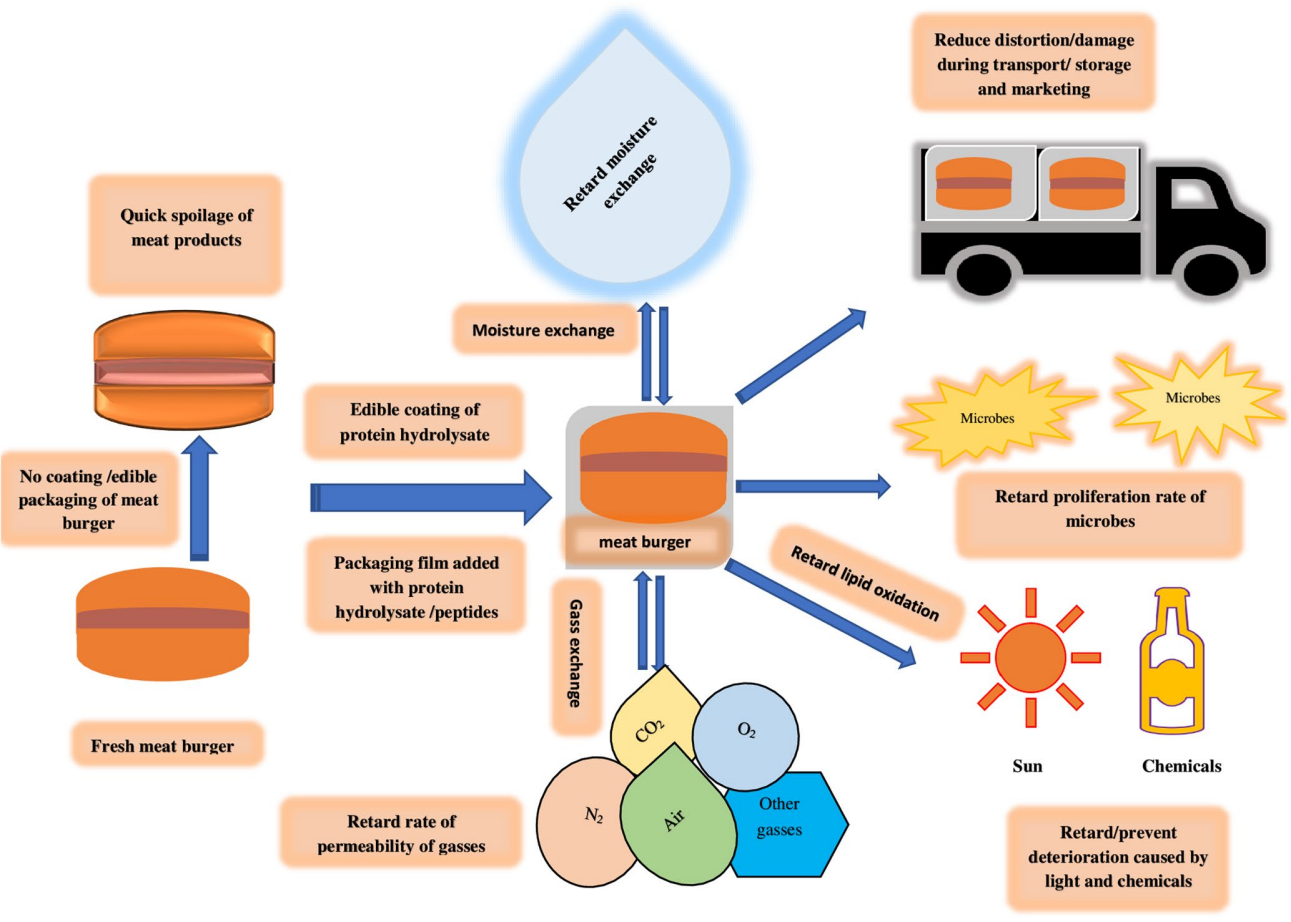
Application of protein hydrolysate/peptides as coating or in packaging materials as active packaging for extension of shelf life of meat and meat products.

Bioactive peptides have also been explored for improving the functionality of packaging materials used for containing and preserving food products (Table 3). They are used as bio‐preservatives due to their good antioxidant and antimicrobial potential. Peptides can be incorporated directly into the formulation for extending the shelf life of products or can be used as components of packaging materials (Figure 4).

**TABLE 3:** Bioactive peptide use as active component in packaging material for preservation of meat and meat products.

Film components	Bioactive peptide/hydrolysate source	Product/enzymes	Functionality	References
Gelatin	Cuttlefish ( *Sepia officinalis* ) skin protein isolate (CSPI) and hydrolysates (CSPH)	Savinase and Purafect	Higher UV‐barrier properties, Higher antioxidant potential	Kchaou et al. (2020)
Bovine gelatin, Smooth‐hound viscera proteins	Smooth‐hound peptides (SHP)	Purafect	Good UV barrier properties, Improved antioxidant properties	Abdelhedi et al. (2018)
Composite films based on chitosan and fish gelatin	Shrimp and crab protein hydrolysates	Bromolein, alcalase, and crude bacterial enzymes from *Bacillus cereus* SV1, *Bacillus subtilis* A26, *Bacillus mojavensis* A21, *Bacillus pumilus* A1, *Bacillus amyloliquefaciens* An6, and *Bacillus licheniformis* NH1	Exhibited higher UV‐barrier properties, improvement in the mechanical properties, and thermal degradation temperatures, greatly enhanced the antioxidant property	Hajji et al. (2021)
Agar films	Fish protein hydrolysate and clove essential oil	Alcalase (A20) and Protamex (P20) Applied on flounder ( *Paralichthys orbignyanus* ) fillets	Increased the water solubility, water vapor permeability, elongation at break and yellowness of the films	Rocha et al. (2018)
Squid skin gelatin films	Giant squid ( *Dosidicus gigas* ) Gelatin hydrolysates	Alcalase	Increased values of FRAP and ABTS, decreased puncture force, increased puncture deformation, and increased water vapor permeability	Giménez et al. (2009)
Bacterial cellulose nanofibers (BCNFs)	Gelatin hydrolysate (GH) from tilapia skin	Alcalase	Antioxidant activity of 7.8 μmols Trolox Eq/g film, a water vapor permeability (WVP) of 1.6 g.mm/kPa.h.m^2^ and an Young's modulus of 0.57 Gpa, Nontoxic	Lima et al. (2018)
Chitosan, xanthan gum	Protein hydrolysate of Whitemouth croaker ( *Micropogonias furnieri* )	Alcalase	Increased antioxidant activity	de Morais Lima et al. (2017)
Fish protein hydrolysates (FPH) and glycerol	Hydrolysate produced from trout byproducts	Conventional enzyme + ultrasound	Higher antimicrobial lower total volatile basic nitrogen (TVB‐N), lower thiobarbituric acid (TBA), lower trimethylamine (TMA) production	Misir and Koral (2019)
Gelatin film	Silver carp protein hydrolysates	Alcalase	Increased DPPH, FRAP, ABTS activity, higher EAB, Lower tensile strength, elastic modulus, L*, b*, and contact angle	Hasanzati Rostami et al. (2017)
Chitosan	Squid gelatin hydrolysates	Alcalase	Higher ABTS and DPPH activity, improved the fungistatic activity against *Aspergillus parasiticus*	Cuevas‐Acuña et al. (2021)
Soy protein isolate; sunflower protein isolate films	Bovine plasma hydrolysate	—	Conferred antioxidant properties, increased elongation at break and water vapor permeability, decreased tensile strength, elastic modulus, and glass transition temperature of the films	Salgado et al. (2011)
Gelatin films	Whitecheek shark ( *Carcharhinus dussumieri* ) protein hydrolysate	Alcalase	Hydrolysate showed linear relation with elongation at break and an inverse relation with the tensile strength and elastic modulus of the films	Alinejad et al. (2017)
Chitosan	Fish protein hydrolysate obtained from frames (skeleton with the meat attached to it) of common carp ( *Cyprinus carpio* )	Flavourzyme Film applied on rainbow trout ( *Oncorhynchus mykiss* ) fillets	Better barrier against lipids oxidation and bacterial proliferation	Reyhani Poul and Jafarpour (2020)
Chitosan coating	Shrimp ( *Litopenaeus vannamei* ) processing residues protein hydrolysates (head and exoskeleton)	Viscozyme/alcalase	Higher antioxidant capacity	Arancibia et al. (2014)

Packaging materials natural or synthetic have been explored as carriers for bioactive peptides. Romani et al. (2020) developed functional polyethylene film packaging material with the ability to scavenge free radicals by conjugating fish protein hydrolysates. Polyethyleneimine was used to tether peptides onto the polyethylene surface, which was later activated by UV‐ozone treatment. Tkaczewska et al. (2021) extended the shelf life of Atlantic mackerel by 2 days with furcellan and carp skin gelatin hydrolysate biopolymer stored at 4°C. The single‐layer and double‐layer polymers efficiently reduced lipid oxidation during storage, controlled microbial proliferation, and did not affect sensorial perception, as perceived by consumers. Bi et al. (2020) identified a functional 16 amino acid peptide, Sm‐A1 (GITDLRGMLKRLKKMK) with significant antibacterial activity against both Gram‐positive and Gram‐negative bacteria. The authors further investigated its potential in the hydrogel of hydroxyl‐rich poly (vinyl alcohol) (PVA)/chitosan (CS). Hydrogel with a 7.5% concentration of Sm‐A1 prevented microbial deterioration of salmon muscle without affecting textural properties.

Various methods or modes of incorporation have been investigated for the utilization of peptides in packaging materials such as direct incorporation in film‐forming material/solution, peptides as coatings, immobilization of active peptides, and encapsulation of peptides in packaging material. Bioactive peptides or hydrolysates can be added directly into the film‐forming solutions to form functional packaging material or film.

Rocha et al. (2018) observed that the incorporation of argentine croaker (fish) hydrolysate in agar films affected its physical characteristics, of which transparency and thickness were not much affected, but tensile strength decreased significantly from 27.46 to 19.89 MPa. The decrease was attributed to the presence of short‐chain peptides in hydrolysates that act as plasticizers and increase free movement by reducing interaction between the polymers, resulting in weaker tensile strength (Nuanmano et al. 2015; Rocha et al. 2018). The plasticizing effect of small chain peptides also affected the water vapor permeability of the agar film by increasing it from 1.40 g mm/h Pa cm^2^ in agar films to 3.61 g mm/h Pa cm^2^ in croaker fish hydrolysate incorporated agar films.

Tkaczewska et al. (2021) developed single‐layer and double‐layered furcellaran/carp skin gelatin hydrolysate coating for the preservation of Atlantic mackerel at refrigeration storage. The film illustrated excellent antimicrobial potential, restricted the development of biogenic amines which even until the end of storage did not exceed 7 mg/kg, and extended the shelf life of mackerel. The same film was evaluated for its film‐forming properties and preservation of Atlantic mackerel at frozen storage by Jamróz et al. (2022). The second functional layer was of Ala‐Tyr dipeptide which did not affect the transparency of the film but decreased its flexibility, decreased the solubility of the film by about 30%, and the tensile strength and elongation at break (%) increased from 8.7 to 13.4 MPa and 28.5% to 37.1% respectively. the second layer of dipeptides increased the elasticity and strength of the film.

The plasticizing effect of fish gelatin hydrolysate was compared to that of glycerol by Nuanmano et al. (2015) in the formation of fish myofibrillar protein film. Gelatin hydrolysate with 23%, 61%, and 95% degree of hydrolysis was incorporated in the film at four different levels: 30%, 40%, 50%, and 60%. The incorporation of hydrolysate decreased the transparency of films due to its interaction with the fish myofibrillar proteins forming a dense network, hindering the transmittance of light. However, hydrolysate with 95% DH evinced significantly transparent films comparable to that of glycerol‐incorporated films at the 60% level of incorporation, which was attributed to the shorter chain length of peptides that affected the protein–protein interaction, reducing the compactness of the film. The plasticizing effect of hydrolysates was more pronounced in hydrolysates with a higher degree of hydrolysis, i.e., 50% and 60%, as they showed higher elastic modulus and tensile strength (MPa). The gelatin hydrolysate showed a good plasticizing effect, which is affected by the degree of hydrolysis.

## 7 Safety and Consumer Acceptability

Food allergy is an adverse immune response that occurs repeatedly toward specific food. The utilization of meat byproduct hydrolysates or peptides might be a promising approach for product formulations and packaging alternatives as natural preservatives, but the toxicological aspect or the allergenicity of such compounds needs attention before commercialization or widespread use. Lafarga et al. (2016) utilized amino acid prediction and motif prediction approaches to identify the toxicity and allergenicity of bovine blood protein hydrolysates prepared with the enzyme papain. Two functional peptide fractions FI and FII were identified with angiotensin‐I‐converting enzyme (ACE‐I) inhibitory and dipeptidyl peptidase‐IV (DPPIV) inhibitory action. Both these fractions were identified as nontoxic in silico studies, but the allergenicity results of the two methods were contradictory. The SVM amino acid approach predicted 152 and 1072 peptides in FI and FII with allergenic potential, while the MEME/MAST predictive approach recorded none.

The studies on the toxicity and allergenicity of bioactive peptides or hydrolysates from animal and/or fish byproducts are limited, but bioactive peptides from other sources have been studied. Fitzgerald et al. (2013) reported the non‐toxicity of seaweed *Palmaria palmata* hydrolysate with platelet‐activating factor acetyl hydrolase (PAF‐AH) inhibitory bioactivity. The toxicity of identified bioactive peptides having the amino acid sequence NIGK was assayed in the zebrafish model, and a concentration of 1 mg/mL was reported to be nontoxic. Canistro et al. (2017) evaluated the toxicity and metabolic effect of rapeseed and sunflower protein hydrolysate in mice models. Both the hydrolysates had good palatability and were well tolerated by mice. They gave comparable results to control in terms of body and organ weight and biochemical blood parameters. The study concluded the nontoxic effect of rapeseed and sunflower protein hydrolysate, which could be used as food ingredients.

Protein hydrolysates of bioactive peptides have shown good functional properties as well as in products for their preservative and positive physiological activities. Toxicological and safety aspects, although meager, have shown nontoxic or safe characteristics of animal byproducts or coproducts hydrolysates/peptides. Laboratory sensory analysis has also shown positive responses for their utilization in products, but large‐scale consumer perception has not yet been thoroughly analyzed to predict the consumer acceptance of these hydrolysates and bioactive peptides. Although Kessler et al. (2019) explored the potential of umami‐tasting meat protein hydrolysates from beef and pork in the formulation of sausages and analyzed their consumer acceptability. A consumer acceptance study was done with 100 volunteers consisting of 61 men and 39 women participants from young and elderly age groups. The incorporation of meat protein hydrolysate did not affect the textural and sensorial properties, and the values of which were similar to the control, and it did not affect the acceptability of products. The acceptability of products with umami amino acids was more governed by the type of recipe rather than the amino acids, which was attributed to the lack of expertise in recognizing tastes by consumers as compared to expert taste panelists.

## 8 Conclusions

Animal or fisheries byproducts are not waste; these are coproducts that are not utilized to their potential due to the technological inefficiency of the producers or processors or rather lack of opportunities. utilization of these coproducts dates back to earlier days of civilization where each and every component of animals killed for food purposes was used. Even today, in many traditional dishes and delicacies, these coproducts are being used, but the portion utilized is far less than the amount disposed of. Disposal leads to its decomposition with an environmental burden, but if the degradation of these coproducts is done in a scientific manner with technological advances, then they might be utilized to produce functional bioactive peptides or hydrolysates. Various studies have established the functional activities of these coproducts. The present research needs are to find out economical and sustainable methods and technologies to harvest this fifth quarter, which is being wasted. With the application of advanced technological and scientific knowledge, the fifth quarter, which lies on the negative scale of investment, might rise to the positive scale and would become a source of revenue.

Recent research has established the functional properties of bioactive peptides and hydrolysates generated from meat and fishery byproducts. These have also been explored as ingredients for product formulations, delivery agents in packaging systems, as alternatives to chemical preservatives, etc. The natural and chemical‐free products market is at an escalating stage, which can be harnessed by the meat processors and industry. These hydrolysates and bioactive peptides from meat coproducts are promising alternatives. Thus, elaborate studies on the safety and toxicological aspects of various hydrolysates/bioactive peptides are required for the actual commercialization of these compounds. More consumer‐scale studies are necessary to understand the acceptability of these novel natural preservatives. To bring these studies to market, a strong governmental willpower will be required. Proper guidelines for safe and healthy levels for incorporation in products and packaging must be decided and declared. Policies regarding SOP for generating such peptides/hydrolysates for food purposes, standard levels, forms of incorporation, types of food items, labeling of such ingredients, components, and categories of consumers where these may be added must be established. Utilization of meat and fishery byproducts or coproducts for the preparation of functional and bioactive peptides and hydrolysates would thus open new avenues for the meat and fishery industry, which would give them the real identity of the fifth quarter.

## Author Contributions

**Akhilesh Kumar Verma:** conceptualization (equal), software (equal), supervision (equal), validation (equal), writing – original draft (equal), writing – review and editing (equal). **Pramila Umaraw:** conceptualization (equal), software (equal), visualization (equal), writing – original draft (equal), writing – review and editing (equal). **Pavan Kumar:** conceptualization (equal), software (equal), writing – original draft (equal), writing – review and editing (equal). **Nitin Mehta:** conceptualization (equal), visualization (equal), writing – original draft (equal), writing – review and editing (equal). **Neelesh Sharma:** conceptualization (equal), resources (equal), supervision (equal), writing – review and editing (equal). **Devendra Kumar:** conceptualization (equal), writing – original draft (equal), writing – review and editing (equal). **Awis Qurni Sazili:** conceptualization (equal), supervision (equal), writing – review and editing (equal). **Sung‐Jin Lee:** funding acquisition (equal), project administration (equal), writing – original draft (equal), writing – review and editing (equal).

## Ethics Statement

The authors have nothing to report.

## Conflicts of Interest

The authors declare no conflicts of interest.

## Data Availability

They are presented in the form of tables and figures.
